# Serogroup-specific Seasonality of Verotoxigenic *Escherichia coli*, Ireland

**DOI:** 10.3201/eid2204.151160

**Published:** 2016-04

**Authors:** Patricia Garvey, Anne Carroll, Eleanor McNamara, André Charlett, Kostas Danis, Paul J. McKeown

**Affiliations:** European Centre for Disease Prevention and Control (ECDC) European Programme for Intervention Epidemiology Training, Stockholm, Sweden (P. Garvey, K. Danis);; Health Service Executive–Health Protection Surveillance Centre, Dublin, Ireland (P. Garvey, P.J. McKeown);; ECDC Public Health Microbiology Training Programme, Stockholm (A. Carroll);; Health Service Executive Public Health Laboratory–Dublin Mid-Leinster, Dublin (A. Carroll, E. McNamara);; Public Health England, London, UK (A. Charlett);; Institut de Veille Sanitaire, Paris, France (K. Danis)

**Keywords:** Shiga-toxigenic Escherichia coli, E. coli, verotoxigenic, VTEC, seasons, seasonality, time series, bacteria, Ireland

**To the Editor:** Globally, an increasing number of serogroups of verotoxigenic *Escherichia coli* (VTEC) have been reportedly associated with human illness. The best known is serogroup O157; the World Health Organization also recognizes VTEC O103, O111, O145, and O26 as having the potential to cause severe disease (*1*). The increasing number of non-O157 VTEC infections is cause for concern. In general, human infections with VTEC are reportedly more common in late summer; the European Centre for Disease Control and Prevention reported that the number of cases across the European Union peaks each year during July–September (*2*). Similarly, the United States reported that the number of VTEC O157 cases peaks in late summer (*3*).

Ireland is now one of the countries with the highest incidence of VTEC infection (*2*). In Ireland, statutory notification of VTEC infection became mandatory in 2004. In common with surveillance internationally, the focus was initially on VTEC O157; since then, testing and surveillance for non-O157 VTEC have improved substantially as a result of increased awareness and availability of diagnostic methods for non-O157 detection. Non-O157 VTEC were first reported in Ireland in 1999 (*4*), and surveillance data indicated that only 14% of VTEC notifications in 2004 compared with 75% in 2014 were caused by non-O157 VTEC. 

In the notification dataset for Ireland, the 2 primary VTEC serogroups (O26 and O157) over many years have seemed to differ in their seasonality; VTEC O26 notifications generally peaked ≈2 months earlier than VTEC O157 notifications (Figure, panel A). This earlier incidence peak for VTEC O26 has become progressively more consistent as the number of reported VTEC O26 notifications has risen. A study by Rivero et al. also suggested that non-O157 human infections may not exhibit the same seasonal variation observed for VTEC O157 (*5*).

**Figure:**
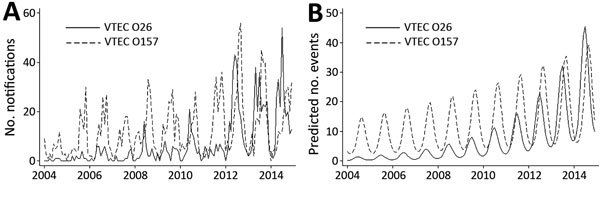
Verotoxigenic *Escherichia coli* (VTEC) O157 and VTEC O26, Ireland, 2004–2014. A) Seasonal distribution of notifications. B) Predicted seasonal distribution. Data source: Computerised Infectious Disease Reporting System (https://www.hpsc.ie/NotifiableDiseases) in Ireland, as of June 24, 2015. Predicted number of cases by month were derived from a cyclical quasi-Poisson model after trend and seasonality and interaction by serogroup were accounted for.

In this study, we compared the seasonality of the 2 strains by using national notification data for 2004–2014 (n = 2,569 notifications for O157 and O26). We estimated the timing of the seasonal peaks (phase of seasonality) for each of the serogroups, and the difference between the 2 phases, by using times series quasi-Poisson regression, fitting terms for temporal trend, and a sine wave with a period of 12 months for seasonality and for interaction by serogroup. We compared the phase shifts of the 2 serogroups by using the Wald test. To rule out the possibility that the observed distributions were influenced by the occurrence of a limited number of outbreaks, we reanalyzed the data for sporadic cases alone and, because risk factors for VTEC infection have been shown to vary by age (*6*), separately for patients <5 years of age and for older child and adult patients.

The number of predicted cases peaked in July for VTEC O26 and in September for VTEC O157; the 2-month difference in phase (seasonality) by serogroup was significant (p<0.0001) (Figure, panel B). The difference in seasonality remained significant (p<0.0001) for sporadic cases alone; the predicted 2-month difference in seasonality was the same. The serogroup-dependent seasonality also remained when the data were analyzed separately for patients <5 years of age (predicted difference in phase 2 months, p<0.0001) and >5 years of age (predicted difference in phase 1 month, p<0.0001).

A significant increasing annual trend was also observed, in particular for VTEC O26. However, this increase is probably, at least in part, artifactual because of increased availability and more widespread use of clinical diagnostic tests for non-O157 VTEC in later years.

One possible explanation for the difference in seasonality is that the primary animal reservoirs for the 2 serogroups could differ. Cattle and sheep have been identified as carriers of O157 and O26 strains in Ireland (*7*,*8*). In Germany, cattle density has been shown to be significantly associated with human VTEC O157 incidence but only marginally associated with O26 incidence (*9*); the same study showed no association between cattle density and VTEC O91 infection, indicating that not all serogroups necessarily share the same reservoirs. Alternatively, animals of the same species may be preferentially colonized with different serogroups at different times of the year or at different developmental ages. Other possible explanations could be variation in survival characteristics between the 2 strains, which results in a different seasonal distribution in the environment, or specific human behavior (e.g., seasonal food) resulting in more frequent exposure to sources of VTEC O157 and VTEC O26 at different times of the year.

The consistent differences in seasonality identified here between the 2 most common VTEC serogroups suggest the existence of noteworthy underlying differences in disease etiology between the strains. Further exploration is recommended.
